# Regorafenib Prior to Selective Internal Radiation Therapy Using ^90^Y-Resin Microspheres for Refractory Metastatic Colorectal Cancer Liver Metastases: Analysis of Safety, Dosimetry, and Molecular Markers

**DOI:** 10.3389/fonc.2019.00624

**Published:** 2019-07-10

**Authors:** Andrew Kennedy, Dianna Shipley, Max Shpak, Laura Blakely, Brian Hemphill, Kent Shih, Cassie Lane, Lisa Zimmerman, Andrew McKenzie, Mark Mainwaring, James D. Peyton, John Zubkus, David Wright, Jaswinder Singh, Johanna C. Bendell

**Affiliations:** ^1^Sarah Cannon Research Institute, Nashville, TN, United States; ^2^Tennessee Oncology, PLLC, Nashville, TN, United States; ^3^Center for Systems and Synthetic Biology, University of Texas, Austin, TX, United States; ^4^Florida Cancer Specialists, Tampa, FL, United States; ^5^HCA Midwest Health, Kansas City, MO, United States

**Keywords:** SIRT, ^90^Y-resin microspheres, colorectal cancer, liver metastasis, radiation therapy

## Abstract

**Background:** This Phase II, open-label, study examined the safety of regorafenib followed by selective internal radiation therapy (SIRT) with regorafenib re-initiation in the treatment of metastatic colorectal cancer (mCRC) patients with liver metastases who are not surgical candidates.

**Methods:** Patients received 160 mg regorafenib daily on a 21-day course followed by a 1 week washout prior to SIRT. Liver function was evaluated at 2 and 4 weeks after SIRT, and regorafenib re-initiated if liver function was normal. Patients were evaluated for safety, and restaged at weeks 6 and 12 following SIRT. In addition, protein and cytokine assays of blood were performed to identify candidate molecular biomarkers associated with outcomes. Individual patient voxel-based dosimetry assessment was performed post-SIRT.

**Results:** Twenty-Five patients were enrolled and received a median 11 weeks regorafenib. Three patients received regorafenib, but not SIRT due to disease progression. The remaining 22 patients received SIRT with a median activity delivered to the liver of 38 mCi, mean normal liver dose of 14.98 Gy and tumor mean dose of 29.0 Gy with a tumor to normal ratio mean of 2.42. There were four treatment-related serious AEs and no treatment-related deaths. Median progression-free survival was 3.7 months and the median overall survival was 12.1 months. The relative densities of several biomolecules changed significantly during the course of treatment, most notably post-treatment increases in levels of sex-hormone binding globulin (*SHBG*) and decreased levels of the cytokine *MIG (CXL9)*. Decreases in von Willebrand factor (*VWF*), the ankyrin repeat domain (*ANKRD26*), and *MIG* were associated with improved survival times. Post-treatment increases in alpha-2-macroglobulin (*A2M*) and the cytokine intercellular adhesion molecule (*ICAM-1)* were associated with reduced overall survival time, while increases in Eotaxin (*CCL14)* predicted longer overall survival times.

**Conclusions:** The treatment of mCRC patients with liver metastases using regorafenib followed by SIRT was tolerable in this patient population. Further efficacy analysis of this treatment schema and analysis of potential molecular biomarkers using larger sample sizes is merited.

## Introduction

It is estimated that 145,600 new cases of colorectal cancer (CRC) will be diagnosed in the United States and ~51,020 patients will succumb to the disease in 2019. Colorectal cancer remains the third most frequently diagnosed cancer in the United States and is responsible for 9% of all cancer deaths (1).

Regorafenib is an oral, multikinase inhibitor with FDA approval for treatment of patients with mCRC who have been previously treated with chemotherapeutic regimens including fluoropyrimidine, oxaliplatin, and irinotecan-based chemotherapy, anti-VEGF therapy, and (if KRAS wild type) with an anti-EGFR therapy. This was based on results from the CORRECT trial, a Phase III randomized, double blinded trial of regorafenib vs. placebo in patients with mCRC who had exhausted all other available treatment options. The CORRECT study showed an improvement in overall survival (OS) of 6.4 vs. 5.0 months (HR 0.77) for patients treated with regorafenib, and the progression free survival (PFS) of patients on regorafenib was 1.9 months vs. 1.7 months for placebo (2).

While recent targeted therapies and treatment strategies have shown promise in CRC, elimination of disease once spread to the liver remains a challenge. Preoperative chemotherapy may improve resectability but may also impact overall survival. Postoperative complications have been associated with decreased long-term survival after surgery for CRC with liver metastases with curative intent (3).

Radioembolization (RE), or selective internal radiation therapy (SIRT), with yttrium-90-labeled (^90^Y) microspheres is a form of brachytherapy that uses radiation damage from locally implanted microspheres (4) to target hepatic tumors while limiting the dose to the liver parenchyma (5). ^90^Y-resin microspheres, 30 microns in diameter, are administered through an intra-vascular catheter inserted in the hepatic artery, entering tumors and permanently lodging in the small intratumoral arteries. Beta radiation emits from the microspheres for about 14 days delivering a large total dose of radiotherapy inside the tumor.

^90^Y-resin microspheres are approved by the Food and Drug Administration (FDA) for the treatment of mCRC that has spread to the liver, and a number of safety and efficacy studies have been published involving patients with mCRC with liver metastases. Radioembolization using ^90^Y-resin microspheres to treat liver-only or liver-dominant mCRC has been successful in this refractory setting (6–8), and combining regorafenib and ^90^Y-RE is an attractive option as an anti-tumor and maintenance treatment for the refractory mCRC population.

We present the safety analysis of the first of what was originally a two-cohort Phase II, open-label study comparing the safety of the combination of SIRT and regorafenib where regorafenib occurs either before or after SIRT. Additionally, we performed exploratory screening for molecular biomarkers that may prove to be predictive of radiation dose response, radioresistance, and patient outcomes using mass spectrometer estimates of peptide abundance and assays for cytokine density. The second cohort was not initiated due to the high probability of insufficient enrollment as a consequence of numerous competing trials.

## Methods

Accrual to this open-label phase 2 trial (NCT01815879) was initiated in July 2014 and was conducted according to the ethical principles of the Declaration of Helsinki and in accordance with the International Conference on Harmonization Guideline for Good Clinical. The study was approved by the institutional review board of all participating sites prior to enrolling patients, and all patients provided informed consent.

### Patients

Eligible patients were required to have histologically confirmed metastatic adenocarcinoma of the colon or rectum, with measurable computed tomography (CT) scan evidence of liver metastases not treatable by surgical resection or local ablation. In addition, patients were required to be appropriate candidates for regorafenib therapy and to have a baseline Eastern Cooperative Oncology Group (ECOG) performance status of 0 or 1 and measurable disease according to the Response Evaluation Criteria in Solid Tumors (RECIST) version 1.1 criteria (9). Adequate baseline hematologic and organ function, defined as absolute neutrophil count (ANC) ≥1,500/μL; hemoglobin ≥9 g/dL [pre-enrollment transfusions were allowed]; platelets ≥75,000/μL; alanine aminotransferase (ALT) and aspartate aminotransferase (AST) <2.5 times the institutional upper limit of normal (ULN), or <5 times ULN; total bilirubin ≤1.5 times ULN [unless patient has bilirubin elevation due to Gilbert's disease]; serum creatinine ≤1.5 mg/dL [133 μmol/L] or calculated creatinine clearance ≥50 mL/min were required for enrollment Patients who had been treated with, or were not candidates for fluorouracil, oxaliplatin, irinotecan, and if KRAS wild-type, anti-EGFR therapy were eligible for enrollment.

### Study Design and Treatment

Patients received 160 mg oral regorafenib daily on days 1–21 of a 28-day cycle. This was followed by a 1 week washout period prior to infusion with ^90^Y-RE (SIR-Spheres® Sirtex, Australia). ^90^Y-resin microspheres were administered in accordance with the terms of its instruction for use and institutional standard of practice. Liver function was evaluated 2 weeks and 4 weeks after SIRT administration and regorafenib was re-initiated at 4 weeks after SIRT if liver function did not have Grade 3 or higher toxicity. Patients were evaluated for safety, and restaged on week 6 and 12 following SIRT treatment and then followed for toxicity and disease progression every 3 months (± 1 month) for up to 6 months after enrollment of the last patient. After documented disease progression, patients were followed for survival every 3 months (± 1 month) for up to 6 months after enrollment of the last patient with laboratory, physical exam, and multiphasic CT scan. The primary objective was to evaluate the safety of this treatment schema.

### Regorafenib Dose Modifications

Dose reductions of regorafenib or holds and initiation of supportive care were allowed as clinically indicated by the treating physician. Patients whose treatment was delayed due to toxicity proceeded with the next cycle of treatment when toxicity had improved as long as the toxicity resolved within 3 weeks. For dosing delays more than 3 weeks, treatment was discontinued. Patients were permitted two dose reductions of regorafenib to manage NCI CTCAE drug-related toxicities ≥Grade 3. For patients who developed ≥Grade 3 hematologic toxicities or Grade 3 non-hematologic AEs, regorafenib was held until recovery to ≤Grade 2 then re-initiated at one dose level lower. Patients who developed Grade 4 non-hematologic toxicity discontinued regorafenib treatment. Liver function tests were monitored throughout the study and patients with Grade 2 or 3 liver-related AEs had regorafenib held until improvement to ≤ Grade 1. All Grade 4 and recurrence of Grade 2 or 3 liver-related AEs resulted in regorafenib discontinuation.

### SIRT Treatment and Dosimetry

Eligibility, patient selection, and procedure details of hepatic artery radioembolization using resin Y90 microspheres is well described (10). Each patient was evaluated and treated with SIRT according to the eligibility requirements and treatment standards outlined in the report (10). Resin Y90 microsphere activity prescriptions were calculated using the BSA method only (11). Patient-specific voxel based 3D dosimetry was accomplished using commercially available software (MIM SurePlan®, Cleveland, OH, USA) (12) of post-Y90 PET scans or SPECT bremsstrahlung gamma scans obtained within 6 h of SIRT treatment as per prior publications (13–15). Dose volume histograms (DVH) were analyzed individually with mean absorbed radiation dose in normal liver and in tumor specifically used in statistical testing for potentially associated factors and outcomes.

### Statistical Plan

There was no formal hypothesis testing in this study. Overall Response Rate (ORR) was defined as the proportion of patients with observed complete response or partial response according to the RECIST 1.1 criteria. Progression-free survival was defined as the interval from first study treatment until objective disease progression or death. Patients who did not have disease progression or death documented were censored on the date of the last visit with an adequate assessment. Overall survival was defined as the interval from first study treatment until death from any cause. Patients who were still alive at the end of study were censored on the date of last contact. Survival estimates were performed using the method of Kaplan and Meier (16). The initial design of the study was to evaluate the safety of the combination of regorafenib and ^90^Y-RE, with one cohort of patients receiving regorafenib prior to ^90^Y-RE treatment, followed by re-initiation of regorafenib, and the other cohort of patients receiving ^90^Y-RE followed by the initiation of regorafenib. However, the study was closed due to slow enrollment and only 1 cohort (patients receiving regorafenib prior to ^90^Y-RE treatment, followed by re-initiation of regorafenib) accrued and treated patients.

### Molecular Assays

To identify changes in biomolecule abundance associated with treatment, the blood serum protein levels were compared over three time points: A- 7 days pre-treatment, B- 8 days post-treatment, and C- 30 days post-treatment. Peptides occurring at high densities were identified from ms/ms spectra provided by Protea Biosciences, following the standard pipeline used to estimate spectral abundance using tandem mass spectrometry (17). Peptide spectrum matches (PSMs) were used to compute spectral abundance factors (SAF = PSM/L, where L is peptide length in amino acids) as an estimate of protein abundance. Because of their lower density, cytokines were assayed using immunochemistry survey panels created by Eurofins (first panel with analytes for 28 cytokines, and two panels with analytes for an additional 4 and 2 cytokines). Cytokine concentrations were estimated from Luminex-quantified optical densities.

The base 2 logarithmic fold change (log FC) between time points B and A, C and A, and C and B was evaluated for each patient as Δ = log_2_[SAF_j_/SAF_i_] where j represents some time point later than i. If multiple replicate samples are available from a single patient's time-point, the arithmetic mean SAF across replicates is used (temporal changes in optical density of cytokines were also quantified as log fold change Δ). By abuse of terminology, we will refer to increased protein levels Δ > 0 as “up-regulation” and decreases Δ < 0 as “down-regulation,” even though changes in protein levels can result from processes other than differential gene expression.

The statistical significance of Δ values' deviations from 0 were evaluated using 2-sided *t*-tests and non-parametric Wilcoxon sign-rank tests for both matched and “pooled” unmatched data. The *p*-values were adjusted using the Benjamin-Hochberg False Discovery Rate (FDR) correction for multiple comparisons. It was assumed that a value of 0 or NA for SAF or optical density represents absence of data rather than an actual concentration of 0. While this creates some bias toward false negatives, it eliminates what would otherwise be a very large set of false positive results. For most ms/ms data, only those proteins with ≥10 samples were retained, while for the cytokines and some of the regression analyses, sample sizes ≥5 were used due to the smaller number of matching samples.

Proteins found to be significantly differentially expressed across treatment time-points were assessed for their functional properties and shared pathways via enrichment analysis using the DAVID gene ontology and functional annotation tool (18, 19). DAVID assigns an enrichment score and *p*-value based on a Fisher exact test of odds ratios by comparing the number of genes in a list associated with a function or pathway to the expected number in that functional role in an equal number of randomly selected genes.

### Molecular Markers for Clinical Outcomes

The association between FC in protein abundance and clinical outcomes was evaluated by regressing patient survival time against treatment time point Δ. Both overall patient survival time (OS) and progression-free survival time (PFS) were regressed against Δ to identify proteins whose FC during treatment predict improved (or poor) patient outcomes. OS values were right-censored because some patients were still alive at the time of last contact. Regression coefficients β > 0 indicate a positive association between up-regulation and increased patient survival (or between down-regulation and reduced survival time), the converse is true for β < 0. The statistical significance of regression coefficients was assessed using FDR-adjusted *p*-values.

### Radiation Absorbed Dose Analysis

To analyze the statistical association between changes in biomolecule abundance and radiation dose, Δ for peptide SAF/cytokine optical density was regressed against mean within-sample tumor radiation dosage (Gy). Similarly, OS and PFS time in patients was regressed against mean tumor dose. Regression analyses were also performed to analyze the association between mean dose with the following blood chemistry metrics: prothrombin (PT) time, ion concentrations (K, Na, Cl, Ca, Mg), concentrations of carcinoembryonic antigen (CEA), glucose, alanine transaminases, blood urea nitrogen, bilirubin, alkaline phosphatase, albumin, creatine, lactate dehydrogenase, and total blood serum protein.

## Results

Between July 2014 and August 2016, 25 patients were enrolled and treated on this trial. Patient characteristics are detailed in Table 1. The median age was 56 years (range: 44–79 years), and 13 patients (52%) were female. Twenty-two patients (88%) had colon tumors and the remaining 3 patients (12%) had rectal cancer. Fourteen patients (56%) had KRAS mutations and the BRAF status for patients was 16 patients (64%) wild-type, 1 patient (4%) mutant, and 8 patients (32%) unknown. Seventeen patients (68%) had prior treatment in the metastatic setting including 8 patients with 1 regimen, 4 patients (16%) with 2 regimens, and 5 patients (20%) with 3 or more prior regimens. Twenty patients (80%) had had prior surgical treatment of the primary tumor.

**Table 1:** Demographics, disease characteristics, and prior therapy.

**Characteristic**	**Total (*N* = 25)**
Median age (range)	56 (44–79)
**Gender**, ***n*** **(%)**	
Female	13 (52)
Male	12 (48)
**Race**, ***n*** **(%)**	
White	22 (88)
Black or African American	3 (12)
**Baseline ECOG**, ***n*** **(%)**	
0	19 (76)
1	6 (24)
**Primary Diagnosis**, ***n*** **(%)**	
Colon	22 (88)
Rectal	3 (12)
**KRAS Status**, ***n*** **(%)**	
Wild-type	14 (56)
Mutant	11 (44)
**BRAF Status**, ***n*** **(%)**	
Wild-type	16 (64)
Mutant	1 (4)
Unknown	8 (32)
Prior Metastatic Regimens, *n* (%)	17 (68)
1 Prior Regimen	8 (32)
2 Prior Regimens	4 (16)
3 or More Prior Regimens	5 (20)
CRC Surgery, *n* (%)	20 (80)

### Treatment

The median duration on treatment was 11 weeks (range 1–86 weeks). Eighteen patients (72%) discontinued due to progressive disease, 4 patients (16%) discontinued due to toxicity or intercurrent events (Grade 2 fatigue, Grade 2 seizure, Grade 4 bowel perforation, and Grade 5 myocardial infarction), and 3 patients (12%) discontinued due to patient decision. Three patients (12%) discontinued prior to ^90^Y-RE administration due to progressive disease. The median activity of ^90^Y-RE delivered to the liver for the 22 patients who received SIRT was 38.2 mCi (Range 1.5–58.5 mCi) and the median lung shunt fraction was 4.3% (range 1.4–13.9%; Table 2). The median tumor volume treated was 75.5 mL (range 5–1,551 mL). Sixteen patients received treatment in the whole liver, 3 patients received treatment in the left lobe, and 3 patients received treatment in the right lobe.

**Table 2:** Patient characteristics.

**Characteristic**	**Total (*N* = 25)**
**Patient received** ^**90**^**Y-resin microspheres**	
Yes	22 (88%)
No^*^	3 (12%)
**Delivered Activity (mCi)**	
*n*	22 pts
Mean	34.4
Standard deviation	15.36
Median	38.2
Minimum	1.5
Maximum	58.5
**Lung Shunt (%)**	
n^**^	21 pts
Mean	4.9
Standard deviation	2.71
Median	4.3
Minimum	1.4
Maximum	13.9
**Treatment Planning**	
*n*	22 pts
Whole	16(64%)
Left and Right	3 (12%)
Right	3 (12%)
**Total Tumor Volume** (mL)	
*n*	21 pts
Mean	177.6
Standard deviation	334.67
Median	75.5
Minimum	5
Maximum	1551

* *3 patients came off study treatment prior to receiving ^90^Y-RE. All 3 patients discontinued due to progressive disease*.

** *Lung shunt unavailable for 1 patient*.

#### Safety

Treatment-related adverse events observed in ≥5% of patients are outlined in Table 3. The most common all grades treatment-related adverse events included fatigue (60%), decreased appetite (36%), nausea (32%), and rash (32%). The most common ≥ Grade 3 treatment-related AEs were hypertension (12%), fatigue (8%), myalgia (8%), palmar-plantar erythrodysesthesia (8%), and hyponatremia (8%). Three patients had treatment-related SAEs: 1 patient had Grade 4 bowel perforation, 1 patient had Grade 3 diarrhea, both SAEs related to regorafenib, and 1 patient had Grade 3 intractable abdominal pain and Grade 3 portal hypertension related to ^90^Y-RE treatment. An additional patient had a Grade 5 myocardial infarct determined to be unrelated to study treatment by an investigator not involved with this study. Seventeen patients (68%) had dose reductions and 20 patients (80%) had dose interruptions. Four patients discontinued treatment due to toxicity or intercurrent events, which included Grade 4 bowel perforation (not close to liver or radiation), Grade 2 fatigue, Grade 2 seizure, and Grade 5 myocardial infarction. No patient experienced radioembolization induced liver disease (REILD) or liver failure.

**Table 3:** Adverse Events (*N* = 25).

**Adverse Events, *n* (%)**	**Grade 1**	**Grade 2**	**Grade 3**	**Total (*N* = 25)**
Fatigue	4 (16)	9 (36)	2 (8)	15 (60)
Decreased appetite	7 (28)	2 (8)	0 (0)	9 (36)
Nausea	5 (20)	3 (12)	0 (0)	8 (32)
Rash	1 (4)	6 (24)	1 (4)	8 (32)
Abdominal pain	1 (4)	5 (20)	1 (4)	7 (28)
Diarrhea	4 (16)	2 (8)	1 (4)	7 (28)
Hyperbilirubinemia	2 (8)	4 (16)	1 (4)	7 (28)
Blister	3 (12)	2 (8)	1 (4)	6 (24)
Mucositis	0 (0)	5 (20)	1 (4)	6 (24)
Vomiting	3 (12)	3 (12)	0 (0)	6 (24)
Arthralgia	2 (8)	3 (12)	0 (0)	5 (20)
Aspartate aminotransferase increased	3 (12)	2 (8)	0 (0)	5 (20)
Constipation	3 (12)	1 (4)	1 (4)	5 (20)
Dysphonia	5 (20)	0 (0)	0 (0)	5 (20)
Headache	4 (16)	1 (4)	0 (0)	5 (20)
Hypertension	1 (4)	1 (4)	3 (12)	5 (20)
Pain in extremity	2 (8)	2 (8)	1 (4)	5 (20)
Thrombocytopenia	4 (16)	1 (4)	0 (0)	5 (20)
Myalgia	0 (0)	2 (8)	2 (8)	4 (16)
Palmar-plantar erythrodysesthesia syndrome	0 (0)	2 (8)	2 (8)	4 (16)
Alanine aminotransferase increased	3 (12)	0 (0)	0 (0)	3 (12)
Anemia	1 (4)	2 (8)	0 (0)	3 (12)
Gastroesophageal reflux disease	1 (4)	2 (8)	0 (0)	3 (12)
Muscle spasms	2 (8)	1 (4)	0 (0)	3 (12)
Pyrexia	1 (4)	2 (8)	0 (0)	3 (12)
Urinary tract infection	1 (4)	2 (8)	0 (0)	3 (12)
Back pain	1 (4)	1 (4)	0 (0)	2 (8)
Dehydration	0 (0)	2 (8)	0 (0)	2 (8)
Dry mouth	1 (4)	1 (4)	0 (0)	2 (8)
Gastrointestinal pain	0 (0)	2 (8)	0 (0)	2 (8)
Hyperkeratosis	1 (4)	1 (4)	0 (0)	2 (8)
Hypokalemia	0 (0)	1 (4)	1 (4)	2 (8)
Hyponatremia	0 (0)	0 (0)	2 (8)	2 (8)
Neutropenia	2 (8)	0 (0)	0 (0)	2 (8)
Tremor	2 (8)	0 (0)	0 (0)	2 (8)
Weight decreased	1 (4)	1 (4)	0 (0)	2 (8)
Dermatitis	2 (8)	0 (0)	0 (0)	2 (8)

#### Y90 Dosimetry

A total of 16 of 22 patients that received SIRT could undergo post-Y90 voxel-based dosimetry calculations. The other six patients did not complete SPECT-gamma (bremsstrahlung) scans post-Y90 as planned due to logistical reasons. Of the sixteen patients, four had PET-Y90 scans, and twelve were SPECT-gamma scans. The normal liver (tumor volume excluded) mean absorbed dose (Gy) from Y90 was 14.98 Gy (9.37–33.94 Gy); mean tumor absorbed dose (Gy) was 29.0 Gy (10.87–64.69 Gy); and the mean Tumor/Normal Liver ratio (T/N) was 2.42 (1.26–5.12).

#### Efficacy

**Figure 1:**
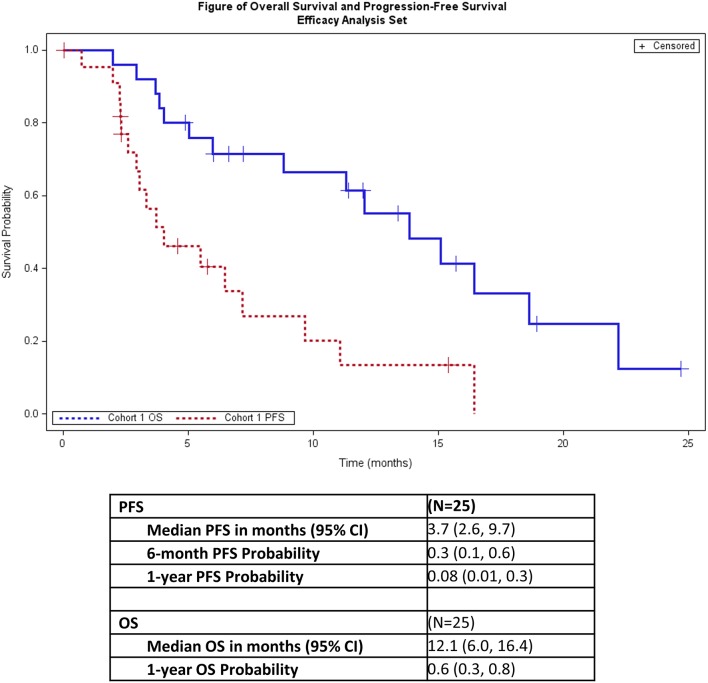
Progression-free survival and overall survival.

One patient (4%) had a partial response and 14 additional patients (56%) had stable disease (Table 4). Seven patients (28%) had progressive disease. Three patients (12%) did not have a post-baseline disease evaluation because the patients withdrew from the study (2 patients) or had clinical progression (1 patient) and thus were deemed unevaluable (Table 4). The overall response rate for all patients was 4% and was 5% for all evaluable patients. The median PFS was 3.7 months (95% CI: 2.6, 9.7), and median OS was 12.1 months (95% CI: 6.0, 16.4; Figure 1).

**Table 4:** Overall response.

**Best Response, *n* (%)**	**Total (*N* = 25)**
Partial response	1 (4)
Stable Disease	14 (56)
Progressive Disease	7 (28)
Unevaluable	3 (12)
Overall Response Rate (all pts)	1 of 25 patients (4%)
Overall Response Rate (evaluable pts only)	1 of 22 patients (5%)

#### Analyses of Biomolecule Levels

For the paired comparisons of ms/ms data, 10–13 patients, depending on the protein, had matched samples. For unmatched comparisons of mean SAFs across time points, the sample sizes range from 12 to 18. For cytokines, the number of patients in all three time points ranges from 5 to 17.

#### Comparison of Peptide SAFs Across Treatment Time-Points

The increase in sex hormone-binding globulin (*SHBG*) between pre- and post-treatment is the only peptide SAF FC that is statistically significant following FDR adjustment (Table 5). The other peptide Δ-values with unadjusted *p* < 0.05 for both up- and down-regulation between treatment time points are shown in Supplement S1. S1 Tables 1a–d do the same for mean comparisons using unmatched SAF values at the three time points. Though not statistically significant post-FDR, the Δ magnitude is large (of the order ~1.0) and *p* << 0.01 for coagulation factor IV (*F9)*, and ceruloplasmin (*CP*), both are down-regulated between pre and post-treatment.

**Table 5:** Proteins (including cytokines) with either statistically significant (post Benjamini-Hochberg FDR adjustment) FC across treatment time points or, qualitatively differential expression based on the criterion of log FC magnitude (|Δ| > 1) across treatment time points.

**UniProt or cytokine name**	**Protein/Gene Name**	**Sample size *n***	**Log FC**	***p***	FDR adjusted p*	**Power (d = 0.2, 0.5, 0.8)**
**P04278**	Sex hormone-binding globulin (*SHBG*)	10	1.887 (C/A)	0.0004	***0.04***	0.09, 0.29, 0.62
P04275	Von Willebrand factor (*VWF*)	11	1.274 (C/B)	0.034	0.52	0.09, 0.32, 0.67
**IP-10**	Interferon gamma-induced protein 10 *(CXCL10)*	9	−1.015 (B/A)	0.018	0.25	0.08, 0.26, 0.56
**MIG**	Chemokine CXC motif ligand 9 (*CXCL9*)	9 7	−1.030 (B/A) −0.681 (C/A)	8.80 ×10^−7^ 1.81 ×10^−6^	***2.73 × 10^−*5*^*** ***5.62 × 10^−*5*^***	0.08, 0.26, 0.56 0.07, 0.20, 0.43

*p-values are based on 2-sided t-test comparison, those in bold also have non-parametric Wilcoxon sign-rank test unadjusted p < 0.05*.

#### Comparison of Cytokine Densities Across Treatment Time-Points

Several cytokines show significantly different densities between treatment time points. MIG (*CXCL9*, chemokine ligand 9) densities decrease significantly (post-FDR) between pre-treatment and post-treatment time points (while increasing between 8 and 30 days). Other cytokine FC with unadjusted *p* < 0.05 are shown in S1 Tables 2a–c.

#### Functional Roles

All 19 proteins with unadjusted *p* < 0.05 based on paired comparison *t*-tests on Δ were included in the DAVID enrichment analysis. As expected for blood serum samples, many of these proteins are characterized as blood plasma and/or secreted, extracellular/exosome peptides. Significant subsets of proteins with large FCs during treatment are involved in complement and coagulation cascades (*C8B, VWF, C4A, SERPINF2, KLKB1, F9*), most of which are down-regulated between pre-and post-treatment. The complete DAVID functional annotation output is summarized in Supplement S2.

#### Molecular Markers of Patient Survival Times

None of the peptide or cytokine treatment course Δ values are significantly associated with patient survival times according to FDR-adjusted p^*^ (Table 6). The lowest *p*-values are for the negative associations between OS vs. Δ von Willebrand Factor (*VWF*), ankyrin repeat domain (*ANKRD26*), and alpha-2-macroglobulin (*A2M*). The decreases in *VWF* and *ANKRD26* levels predict increased OS, while the increases in *A2M* are associated with shorter OS. S1 Tables 3a–c, 4a–c show all regression coefficients with unadjusted *p* < 0.05 between peptide ΔSAF and PFS/OS, respectively.

**Table 6:** Regression analysis FC vs. Survival Time.

**Uniprot or cytokine name**	**Protein/Gene**	**Sample size *n***	**Log FC**	**Survival time**	****β****	***p***	***p***
P01023	Alpha-2-macroglobulin (*A2M*)	10 (B/A)	0.157	OS	−222.58	0.0042	0.22
P04275	von Willebrand Factor (*VWF*)	10 (B/A)	−0.593	OS	−106.40	0.0030	0.22

β is the regression coefficient, regression coefficients, the p-values are based on t-tests evaluating the difference between **β** and 0. p-values are of the order ~0.001 are shown, as no FDR-adjusted p* are < 0.05.

The strongest associations between cytokine density FC and survival times is for OS vs. ΔMIG (chemokine ligand 9, *CXCL9*). This cytokine decreases in density from pre-treatment and post-treatment time points, these changes are negatively associated with OS—i.e., stronger down-regulation corresponds to longer overall survival. S1 Tables 5a,b, respectively, show associations between Δ cytokine and PFS/OS with regression coefficients *p* < 0.05.

#### Radiation Dose and Molecular Markers, Biochemical Markers

Several protein and cytokine Δ values are correlated with mean radiation dosage to the tumor; none of these associations have p^*^ < 0.05 (Table 7). The strongest associations between FC in peptide SAF and tumor radiation dosage are for pre-treatment and 8 days post-treatment time points for sex hormone binding globulin (*SHBG*), which is down-regulated and negatively associated with radiation dose, and cadherin-5 (*CDH5*), which is up-regulated and positively associated with radiation dose. S1 Tables 6a–c shows all associations between SAF FC and radiation dosage with *p* < 0.05 for regression coefficients. Similar patterns are seen for cytokine density FC vs. radiation dose to the tumor (S1 Table 7). MIG levels are positively associated with radiation dosage across both pre-treatment and the 8 vs. 30 day post-treatment time points with unadjusted *p* = 0.02.

**Table 7:** Regression coefficients β for changes in protein density (log FC) across time points against mean dosage (Gy) to tumor.

**Protein accession**	**Protein (Gene name)**	**Mean Log FC**	**Mean tumor dose (Gy)**	**β (log FC protein vs. mean tumor dose)**	***p*-value**	**Adjusted *p*-value**
P04278	Sex hormone binding globulin (*SHBG*)	−0.340 (B/A)	40.02	−0.0619	0.0062	0.32
P33151	Cadherin-5 (*CDH5*)	−0.529 (B/A)	42.57	0.0206	0.0021	0.27

*p-values (based on a t-test evaluating the difference of **β** from 0) are adjusted via Benjamini-Hochberg FDR. Tumor dosage varies due to differences in missing sample points*.

Several biochemical markers have changes correlated with radiation dose. The total amount of bilirubin (mg/dL) has a regression coefficient of −0.01 with respect to radiation dose with an FDR-adjusted p^*^ < 0.005. Other blood chemistry markers, such as levels of alkaline phosphatase and potassium, show large FC in response to high radiation dose, although post FDR p^*^ > 0.05 (Table 8).

**Table 8:** Regression analysis of blood chemistry measures against mean radiation dosage to tumor.

**Blood chemistry**	**Model**	**Mean at screening**	**Summary statistic**	**β**	***p***	p*
CEA	Change vs. Dosage Ratio	35.54 (ng/mL)	Change = 15.67	−202.15	0.046	0.61
Alkaline Phosphatase	Difference vs. Dosage	158.21 (U/L)	Difference = 22.35	−3.02	0.012	0.065
SGOT/AST	Difference vs. Dosage Ratio	41.5 (U/L)	Difference = 5.25	−0.748	0.018	0.30
*Total Bilirubin*	*Difference vs. Dosage*	*0.6 (mg/dL)*	*Difference = 0.18*	*−0.01*	*0.006*	*0.048*
Potassium	Difference vs. Dosage	4.62 (mmol/L)	Difference = −0.37	−0.028	0.039	0.17

*β is the estimated regression coefficient, p-values are based on a t-test of the deviation of β from 0*.

*Change = Last Treatment – First Screening*.

*Difference = Mean Treatment – Mean Screening*.

#### Radiation Dose and Clinical Outcomes

Mean radiation dose to the tumor is weakly predictive of mean PFS time (regression coefficient = 32.93, *p* = 0.052) but not of OS time (which is actually negatively, though not significantly, associated with dose—i.e. β = −0.038, *p* = 0.932). There is a mean decrease in tumor size of 8.38 mm, the regression coefficient (−0.099) between tumor size change and dose is not statistically significant (*p* = 0.60) (S1 Table 8).

## Discussion

The addition of regorafenib to ^90^Y-RE administration was safe and tolerable in patients with metastatic colorectal cancer who were not candidates for surgery. The most common treatment-related adverse events were fatigue, decreased appetite, nausea and rash and were mild to moderate in severity. Most patients were able to tolerate the combination treatment with only 4 patients (16%) withdrawing due to toxicity. This is consistent with data from the phase 3 CORRECT trial and the observational regorafenib study, CORRELATE (20, 21).

One major concern with the addition of regorafenib to ^90^Y-RE therapy is the potential for liver toxicity. While an increase in bilirubin, aspartate aminotransferase, and alanine aminotransferase was seen in these patients, there was only 1 ≥ Grade 3 event (Grade 3 hyperbilirubinemia) and no liver function related SAEs or treatment discontinuations.

Even though efficacy analysis was not the primary objective of this study, response was similar to that seen in other studies (6–8, 22–24). The MORE study which examined SIRT for the treatment of liver metastases had an ORR rate per RECIST v1.1 at 3 months of 6.9 and 48.1% of patients with stable disease. This study had an ORR of 4 and 56% of patients with stable disease. Regorafenib was first approved for the treatment of mCRC based on an improvement of OS from 4 to 6.7 months (2). The OS in this study was 12.1 months with a 1 year OS probability of 60%.

The recent SIRFOX study examined the safety and efficacy of adding ^90^Y-RE administration prior to a FOLFOX6m ± bevacizumab treatment regimen in patients with mCRC with liver metastases who had no previous therapy. This study did not show significant improvement in initial patient response and more ≥ Grade 3 AEs, and liver-related toxicity for patients with the combination therapy (25). It was hoped this result would translate to improvement in OS, but a recent combined analysis of FOXFIRE, SIRFLOX, and FOXFIRE-Global studies showed this not to be the case (26). However, all 3 studies were in the first line setting and SIRT has been shown to be effective with later treatment in a second or third line setting following first line chemotherapy regimens (6, 22). Currently regorafenib is approved for treatment of a similar, later-therapy line, patient population. The combination of ^90^Y-RE with other treatment modalities including regorafenib has the potential to improve efficacy without a significant increase in toxicity in patients with refractory mCRC liver metastases.

This study also identified biomolecules whose blood serum levels correlate with treatment and patient outcomes. Some may serve as biomarkers of treatment course and response, e.g., sex hormone binding globulin (*SHBG*) is strongly up-regulated during the course of treatment. *SHBG* is synthesized in the liver and has an important role in modulating sex hormone levels (27); it is up-regulated in the blood serum of both colorectal (28) and gastric cancer (29) patients. *SHBG's* up-regulation in this group of patients may reflect improvement of liver function in response to reduction in tumor mass. Similarly, the strong down-regulation of the cytokines *CXCL9* and *CCL27* post-treatment may indicate a reduced inflammation response during therapy.

Complement factor H related gene 5, *CFHR5*, whose post-treatment downregulation shows a strong association with increased PFS times, is highly expressed in liver cells, while its allelic variants show statistical association with nephropathies (30, 31). The most robust molecular predictors of OS are the pre vs. post treatment FCs for von Willebrand factor (*VWF*), a blood glycoprotein involved in hemostasis. Its down-regulation during treatment is predictive of improved PFS, consistent with the results of Yang et al. (32), showing that promotion of metastases by *VWF*.

There is no overall pattern to associations between differential expression of cytokines and patient survival times. For some cytokines, up-regulation is associated with improved survival (e.g., Eotaxin/*CCL14*), in others down-regulation is associated with longer survival times (e.g., TNFα). For MIG (*CXLC9*), the negative association between patient survival time and FC may seem surprising in view of the fact that CXCL9 up-regulation has been linked to improved survival in patients with other common cancer types, including breast and ovarian cancer (33). However, the down-regulation in *CXCL9* may simply reflect high values pre-treatment which decline in response to chemotherapy and radiation. Similarly, in MIG, Δ_AB_ and Δ_AC_ < 0 while Δ_BC_ > 0 (S1 Tables 2, 5), the decrease in MIG levels between 8 and 30 days may account for the sign changes the regression coefficient of survival times vs. FC.

Unfortunately, most associations observed between patient outcomes and peptide/cytokine FC are not statistically significant following FDR. This reflects small sample sizes and low statistical power. Future analyses enrolling more patients and using other assays, such as RNASeq for more direct estimates of gene expression levels during the course of treatment may reveal additional, stronger associations between clinical outcomes and molecular profiles.

The ability to acquire accurate and clinically efficient calculations of absorbed radiation dose after Y90 radioembolization has been a significant limitation of this treatment approach until the last few years when commercial software and discovery of time of flight (TOF) PET-based Y90 dosimetry became widely available (13, 34). It is now accepted that the use of TOF PET-Y90 dosimetry provides accurate DVH estimates in normal liver tissue and malignant tumors, including mCRC hepatic lesions (13, 15, 23). Prospective studies suggest TOF PET-Y90 dosimetry reveals a dose-response relationship between absorbed dose of resin Y90 SIRT and mCRC lesions (13, 23). A threshold dose for objective partial response is a minimum dose of 40−60 Gy in tumor (15). One study noted a strong statistically significant dose-effect of resin Y90 microspheres with improved hepatic tumor PFS and OS, with mean tumor dose of 51 Gy (± 28 Gy), range 7–174 Gy (15). In a prospective study of 21 mCRC patients receiving resin Y90 SIRT employing SPECT/CT for post-SIRT dosimetry, the tumor/normal ratio of absorbed dose was significantly (*p* < 0.001) associated with radiographic response (RECIST 1.1), longer PFS and OS in those patients with a T/N ratio of at least 1.7 or tumor dose of 70 Gy (14). Our patients generally received a lower mean tumor dose of Y90 than these studies which might be a factor in the comparatively lower response rate. Hepatic artery patency and tumor hypervascularity are key factors in the ability to deliver adequate Y90 dose to mCRC lesions and remain variables of which physicians cannot manipulate. Subjective observations of hepatic arteries during hepatic angiography suggest changes in vasculature by regorafenib which might have impacted the ability to deliver all of the planned ^90^Y activity to tumors.

The combination of regorafenib with ^90^Y-RE merits further investigation. Further studies of this combination with further analysis of safety and efficacy would be beneficial.

## Data Availability

All datasets generated for this study are included in the manuscript and/or the Supplementary Files.

## Ethics Statement

This study was carried out in accordance with the recommendations of the Western Institutional Review Board (WIRB) and the institutional review board of all participating sites with written informed consent from all subjects. All subjects gave written informed consent in accordance with the Declaration of Helsinki. The protocol was approved by the Western Institutional Review Board (WIRB) and the institutional review board of all participating sites.

## Author Contributions

Study design was developed by AK, MS, and JB. The study was conducted with input from all authors. Data analysis was conducted by AK, MS, LZ, AM, and JB. The manuscript was written with input from all authors.

### Conflict of Interest Statement

JB reports the possible conflicts of interest. Institutional funding by Sirtex, Gilead, Genetech/Roche, Bristol Myers Squib, Five Prime, Lilly, Merck, MedImmune, Celgene, EMD Serono, Taiho, Marcogenics, GSK, Novartis, OncoMed, LEAP Therapeutics, Astrazeneca, BI, Daiichi Sankyo, Bayer, Incyte, Apexigen, Koltan, SynDevRx, Forty Seven, Abbvie, Array, Onyx, Sanofi, Takeda, Eisai, Celldex, Agios, CytoMx, Nektar, ARMO, Boston Biomedical, Ipsen, Meerimack, Tarveda, Tyrogenex, Oncogenex, Marshall Edwards, Pieris, Mersana, Calithera, Blueprint, Evelo, FORMA, Merus, Jacobio, Effector, Novocure, Arrys, Tracon, Sierra, Innate, Arch Oncology, Prelude Therapeutics, Phoenix Bio, Unum Therapeutics, Vyriad, Harpoon, Cyteir, Molecular Partners, ADC, Torque, Tizona, Janssen, Tolero, TD2 (Translational Drug Development), Amgen, Seattle Genetics, Molecular Therapeutics, Tanabe Research Laboratories, Beigene, Continuum Clinical, Cerlean, Pfizer, Millennium, Imclone, Acerta Pharma, and Rgenix. The remaining authors declare that the research was conducted in the absence of any commercial or financial relationships that could be construed as a potential conflict of interest.
