# Supplementary material for: Regorafenib Prior to Selective Internal Radiation Therapy Using 90Y-Resin Microspheres for Refractory Metastatic Colorectal Cancer Liver Metastases: Analysis of Safety, Dosimetry, and Molecular Markers
Source: Front Oncol. 2019 Jul 10;9:624. doi: 10.3389/fonc.2019.00624 (PMC6636394; doi:10.3389/fonc.2019.00624)
Supplement: Supplementary file 1 [file Data_Sheet_1.docx]

**Tables Summarizing Statistically Significant Biomarkers in GI 189 Study**

**S1 Table 1**. Proteins with statistically significant log fold change across treatment time points, based on a t-test comparison of log SAF values across two of the time points (*p* is the p-value of the t-statistic, *p** is the Benjamini-Hochberg adjusted value). Proteins whose log FC across two sample time points are significantly different from 0 (unadjusted *p* < 0.05) according to the non-parametric Wilcoxon sign-rank test in addition to the t-test are highlighted in bold.

**S1 Table 1a:** Comparison of SAF values between time points B (8 days post-treatment) and A (pre-treatment) Positive Log FC indicate an increase in SAF values - up-regulation – at time point C relative to A, while negative values indicate down-regulation in time point C relative to A. Minimum sample sizes for Power = 0.8 (for a significance level α=0.05) at standard deviation fractions *d* = 0.2, 0.5, 0.8 are 199, 34, 15, respectively. The last column shows the statistical power for *d*.

| ***UniProt Name*** | ***Protein/Gene Name*** | ***Sample size n*** | ***Log FC (B/A)*** | ***p*** | ***p**** | ***Power (d=0.2,0.5,0.8)*** |
| --- | --- | --- | --- | --- | --- | --- |
| **P00450** | Ceruloplasmin (*CP*) | 13 | -0.294 | 0.0074 | 0.64 | 0.10,0.38,0.75 |
| P02787 | Serotransferin (*TF*) | 12 | -0.941 | 0.033 | 0.67 | 0.10,0.35,0.71 |
| P05452 | Tetranectin (*CLEC3B*) | 13 | -0.566 | 0.033 | 0.67 | 0.10,0.38,0.75 |
| **P27918** | Properdin (*CFP*) | 12 | -0.361 | 0.041 | 0.67 | 0.10,0.35,0.71 |
| **P06276** | Cholinesterase (*BCHE*) | 12 | -0.802 | 0.030 | 0.67 | 0.10,0.35,0.71 |
| **Q6UY11** | Protein delta homolog 2 (*DLK2*) | 10 | 0.873 | 0.011 | 0.64 | 0.09,0.29,0.62 |
| **A0A075B6Z2** | T cell receptor alpha joining 56 (*TRAJ56*) | 13 | 0.294 | 0.016 | 0.65 | 0.10,0.38,0.75 |

**S1 Table 1b:** Tests for SAF FC between time point C (30 days post-treatment) and A (pre-treatment). N =13

| ***UniProt Name*** | ***Protein/Gene Name*** | ***Sample size n*** | ***Log FC (C/A)*** | ***p*** | ***p**** | ***Power (d=0.2,0.5,0.8)*** |
| --- | --- | --- | --- | --- | --- | --- |
| **H0YAC1** | Plasma kallikrein (*KLKB1*) | 13 | -0.348 | 0.046 | 0.60 | 0.10,0.38,0.75 |
| **P08697** | Alpha-2-antiplasmin (*SERPINF2*) | 12 | -0.244 | 0.017 | 0.51 | 0.10,0.35,0.71 |
| **P01019** | Angiotensinogen (*AGT*) | 13 | -0.289 | 0.031 | 0.60 | 0.10,0.38,0.75 |
| P02753 | Retinol-binding protein 4 (*RBP4*) | 12 | -0.424 | 0.047 | 0.60 | 0.10,0.35,0.71 |
| **P00740** | Coagulation factor IV (*F9*) | 12 | -0.404 | 0.0022 | 0.13 | 0.10,0.35,0.71 |
| **P04278** | Sex hormone-binding globulin (*SHBG*) | 10 | 1.887 | 0.0004 | *0.04* | 0.09,0.29,0.62 |
| **Q9UGM5** | *Fetuin-B* (*FETUB*) | 13 | 0.764 | 0.010 | 0.38 | 0.10,0.38,0.75 |
| **A0A0G2JPR0/**  A0A140TA32 | Complement C4-A (*C4A*) | 13 | -0.078 | 0.042 | 0.60 | 0.10,0.38,0.75 |

**S1 Table 1c:** Tests for SAF FC between time point C (8 days post-treatment) and B (30 days post-treatment). N=13

| ***UniProt Name*** | ***Protein (Gene Name)*** | ***Sample size n*** | ***Log FC (C/B)*** | ***p*** | ***p**** | ***Power (d=0.2,0.5,0.8)*** |
| --- | --- | --- | --- | --- | --- | --- |
| P04275 | Von Willebrand factor (*VWF*) | 11 | 1.274 | 0.034 | 0.52 | 0.09,0.32,0.67 |
| P07358 | Complement component C8 beta chain (*C8B*) | 11 | 0.228 | 0.0073 | 0.44 | 0.09,0.32,0.67 |
| P04196 | Histidine-rich glycoprotein (*HRG*) | 11 | 0.313 | 0.022 | 0.44 | 0.09,0.32,0.67 |
| Q9BXR6 | Complement factor H-related protein 5 precursor (*CFHR5*) | 10 | -0.404 | 0.020 | 0.44 | 0.09,0.29,0.62 |
| P61769 | Beta-2 microglobulin precursor (*B2M*) | 10 | 0.384 | 0.017 | 0.44 | 0.09,0.29,0.62 |

Note: For P03951 (Coagulation factor XI/*F11*), the t-test statistic is not significant, while the Wilcoxon test statistic 39.5 has *p* = 0.05 (adjusted p-value = 1).

**S1 Table 1d:** This table summarized the results of t-tests and Wilcoxon tests performed on pooled, unpaired log-scaled SAF values in time points A,B,C (e.g. including cases where only a single time point is available for a patient). As in the previous examples, t > 0 indicates up-regulation in the later time points. The UniProt names of those that also have *p* < 0.05 for the Wilcoxon tests are in bold.

The statistical power shown in the last column as in Tables a-c. The required sizes of the second sample n_2_ to achieve Power = 0.8 at d = 13, 14, 18, are n_2_ effectively infinite for standard deviation fractions *d* = 0.2 and 0.5. For d = 0.8, the required sample sizes are 249, 113, 43 for the corresponding values of *d*.

| ***UniProt Name*** | ***Protein (Gene Name)*** | ***Comparison*** | ***Sample sizes n*** | ***Mean difference log SAF*** | ***p*** | ***p**** | ***Power (d=0.2,0.5,0.8)*** |
| --- | --- | --- | --- | --- | --- | --- | --- |
| **P00450** | Ceruloplasmin (*CP*) | B/A | 14,18 | -0.291 | 0.030 | 0.99 | 0.08,0.27,0.58 |
| **P05452** | Tetranectin (*CLEC3B*) | B/A | 14,18 | -0.662 | 0.038 | 0.999 | 0.08,0.27,0.58 |
| P04278 | Sex hormone-binding globulin (*SHBG*) | C/A  C/B | 13,14  14,14 | 1.415  0.977 | 0.0002  0.036 | 0.26  0.99 | 0.08,0.24,0.51  0.08,0.25,0.53 |
| **P04196** | Histidine-rich glycoprotein (*HRG*) | C/B | 14,14 | 0.554 | 0.029 | 0.99 | 0.08,0.25,0.53 |

**S1 Table 2**. Comparison of cytokine optical densities across time points. Because most patients are represented with samples from all 3 time points, only the paired comparisons are considered.

**S1 Table 2a:** Cytokine densities for treatment time points B vs. A. Sample size n = 9, statistical power for *d* = 0.2, 0.5, 0.8 are 0.08, 0.26, 0.56, respectively. Minimum sample size to achieve for Power = 0.8 at significance level α = 0.05 is 14.

| ***Cytokine Abbreviation*** | ***Cytokine Name*** | ***Log FC (B/A)*** | ***p*** | ***p**** |
| --- | --- | --- | --- | --- |
| **CTACK** | Chemokine (C-C motif) ligand 27 (*CCL27*) | -0.378 | 0.031 | 0.25 |
| **IP-10** | Interferon gamma-induced protein 10 *(CXCL10)* | -1.015 | 0.018 | 0.25 |
| **MIG** | Chemokine CXC motif ligand 9 (*CXCL9*) | -1.030 | 8.80 x 10^-7^ | *2.73 x 10^-5^* |
| **PDGF** | Platelet-derived growth factor | 0.580 | 0.038 | 0.25 |
| **VCAM-1** | Vascular cell adhesion molecule 1 | -0.359 | 0.042 | 0.25 |
| TNF | Tumor necrosis factor alpha | -3.136 | 0.049 | 0.252 |

**S1 Table 2b:** C vs. A, N = 7, Power for *d* = 0.2, 0.5, 0.8 is P = 0.07,0.20, 0.43.

| ***Cytokine Abbreviation*** | ***Cytokine Name*** | ***Log FC (C/A)*** | ***p*** | ***p**** |
| --- | --- | --- | --- | --- |
| **CTACK** | Chemokine (C-C motif) ligand 27 (*CCL27*) | -0.300 | 0.016 | 0.15 |
| IL-8 | Interleukin 8 (*CXCL8*) | 0.353 | 0.024 | 0.19 |
| **MIG** | Chemokine (CXC motif) ligand 9, (*CXCL9*) | -0.681 | 1.81x10^-6^ | *5.62x10^-5^* |
| **ICAM-1** | Intercellular Adhesion Molecule 1 | 0.524 | 0.0046 | 0.071 |

**S1 Table 2c:** C vs. B, N = 5. Power at *d* = 0.2,0.5, 0.8 is P = 0.06, 0.14, 0.28.

| ***Cytokine Abbreviation*** | ***Cytokine Name*** | ***Log FC (C/B)*** | ***p*** | ***p**** |
| --- | --- | --- | --- | --- |
| EGF | Epidermal growth factor | -0.803 | 0.010 | 0.062 |
| **Eotaxin** | C-C motif chemokine 14 (*CCL14*) | -0.375 | 0.0075 | 0.058 |
| **GRO** | Chemokine ligand 1 (*CXCL1*) | -0.582 | 0.0049 | 0.051 |
| **IL-8** | Interleukin 8 (*CXCL8*) | 0.274 | 0.024 | 0.11 |
| **IP-10** | Interferon gamma-induced protein 10 *(CXCL10)* | 0.825 | 0.0029 | 0.051 |
| MIG | Chemokine (CXC motif) ligand 9 (*CXCL9*) | 0.407 | 0.042 | 0.16 |
| **SCF** | Stem Cell Factor | -0.474 | 0.0470 | 0.16 |
| **ICAM-1** | Intercellular Adhesion Molecule 1 | 0.546 | 0.0037 | 0.051 |
| **VCAM-1** | Vascular cell adhesion molecule 1 | 0.525 | 0.0170 | 0.088 |

**S1 Table 3.** Regression analysis of the association between progression-free survival time (PFS) for a patient and the observed log fold change in SAF between two time points. ***β*** is the regression coefficient, the p-values are for a t-test of the difference of ***β*** from 0. The mean log FC is shown in order to indicate whether the regression coefficient is associated with up- or down regulation of the gene:

**S1 Table 3a:** Time points A and B

| ***Uniprot Name*** | ***Protein/Gene*** | ***Sample Size N*** | ***Log FC (B/A)*** | ***β*** | ***p*** | ***p**** |
| --- | --- | --- | --- | --- | --- | --- |
| P01009 | Alpha-1-antitrypsin (*SERPINA1*) | 10 | 0.761 | -61.90 | 0.029 | 0.71 |
| A0A075B6Z2 | T cell receptor alpha joining 56 (*TRAJ56*) | 13 | 0.295 | 255.47 | 0.020 | 0.71 |
| A0A0B4J1X2 | Voltage dependent t-type calcium channel subunit alpha-1G (*CACNA1G*) | 6 | -0.561 | 197.23 | 0.016 | 0.71 |
| Q9BXR6 | Complement factor H-related protein 5 (*CFHR5*) | 11 | -0.007 | -163.08 | 0.021 | 0.71 |
| P00738 | Haptoglobin (*HP*) | 8 | 1.068 | -128.73 | 0.024 | 0.71 |
| K7EP00 | Protein phosphatase slingshot homolog 2 (*SSH2*) | 7 | 0.202 | 76.95 | 0.025 | 0.71 |

**S1 Table 3b:** Time points A and C

| ***Uniprot Name*** | ***Protein/Gene*** | ***Sample Size N*** | ***Log FC (C/A)*** | ***β*** | ***p*** | ***p**** |
| --- | --- | --- | --- | --- | --- | --- |
| P05115-3 | alpha-2 (*RALGAPA2*) | 7 | 0.0580 | -163.10 | 0.025 | 0.99 |
| Q9BXR6 | Complement factor H-related protein 5 (*CFHR5*) | 11 | -0.1644 | -116.88 | 0.0035 | 0.70 |
| H3BQT6 | Tyrosine-protein phosphatase non-receptor type 9 (*PTPN9*) | 7 | 0.0836 | 420.20 | 0.019 | 0.99 |
| P55291 | Cadherin-15 (*CDH15*) | 7 | 0.0836 | 364.13 | 0.008 | 0.76 |

**S1 Table 3c:** Time points B and C

| ***Uniprot Name*** | ***Protein/Gene*** |  | ***Log FC (C/B)*** | ***β*** | ***p*** | ***p**** |
| --- | --- | --- | --- | --- | --- | --- |
| C9JJ19 | Mitochondrial 28 S ribosomal protein S34 (*MRPS34*) | 9 | -0.392 | 176.20 | 0.035 | 0.92 |
| Q9BXR6 | Complement factor H-related protein 5 (*CFHR5*) | 10 | -0.404 | -257.66 | 0.030 | 0.92 |
| H0YAC1 | Plasma kallikrein (*KLKB1*) | 11 | -0.085 | 174.15 | 0.045 | 0.92 |

**S1 Table 4**. Association between protein SAF log fold change between two time points and patient overall survival time (OS), which has been right-censored to exclude patients who were still alive at the end of the study period. ***β*** is the regression coefficient, the p-values are for a t-test of the difference of ***β*** from 0.**S1 Table 4a**: Time points A and B

| ***Uniprot Name*** | ***Protein/Gene*** | ***Sample Size N*** | ***Log FC (B/A)*** | ***β*** | ***p*** | ***p**** |
| --- | --- | --- | --- | --- | --- | --- |
| P01023 | Alpha-2-macroglobulin (*A2M*) | 10 | 0.157 | -222.58 | 0.0042 | 0.22 |
| P04275 | von Willebrand Factor (*VWF*) | 10 | -0.593 | -106.40 | 0.0030 | 0.22 |
| Q9UPS8 | Ankyrin repeat domain-containing protein 26 (*ANKRD26*) | 7 | -0.313 | -188.84 | 0.0045 | 0.22 |
| P01042 | Kininogen-1 (*KNG1*) | 8 | 0.132 | -471.16 | 0.041 | 0.87 |
| P02775 | Platelet basic protein (*PPBP*) | 10 | -0.404 | -74.05 | 0.023 | 0.66 |
| A0A087WVQ6 | Clathrin heavy chain (*CLTC*) | 6 | 0.005 | 847.13 | 0.0110 | 0.41 |

**S1 Table 4b**: Time points A and C

| ***Uniprot Name*** | ***Protein/Gene*** | ***Sample Size N*** | ***Log FC (C/A)*** | ***β*** | ***p*** | ***p**** |
| --- | --- | --- | --- | --- | --- | --- |
| P01031 | Complement C5 (*C5*) | 8 | 0.220 | -363.66 | 0.016 | 0.71 |
| Q92954 | Proteoglycan 4 (*PRG4*) | 8 | -0.115 | -324.85 | 0.046 | 0.71 |
| P36955 | Pigment epithelium-derived factor (*SERPINF1*) | 8 | -0.013 | -501.08 | 0.026 | 0.71 |
| P07357 | Complement component C8 alpha chain (*C8A*) | 8 | 0.134 | -535.85 | 0.022 | 0.71 |
| P22352 | Glutathione peroxidase 3 (*GPX3*) | 8 | 0.073 | -209.23 | 0.017 | 0.71 |

**S1 Table 4c**: Time points B and C

| ***Uniprot Name*** | ***Protein/Gene*** | ***Sample Size N*** | ***Log FC (C/B)*** | ***Regression Coefficient*** | ***p-value*** | ***Adjusted p-value*** |
| --- | --- | --- | --- | --- | --- | --- |
| P08603 | Complement factor H (*CFH*) | 8 | 0.0048 | 518.38 | 0.030 | 0.61 |
| P19827 | Inter-alpha-trypsin inhibitor heavy chain H1 (*ITIH1*) | 8 | -0.0471 | 551.54 | 0.044 | 0.61 |
| P01042-2 | Kininogen-1 (*KNG1*) | 8 | -0.0348 | 383.82 | 0.032 | 0.61 |
| P02649 | Apolipoprotein E (*APOE*) | 8 | -0.142 | -156.39 | 0.045 | 0.61 |
| P51884 | Lumican (*LUM*) | 8 | 0.183 | 338.99 | 0.0038 | 0.23 |
| P04217 | Alpha-1B-glycoprotein (*A1BG*) | 8 | 0.0014 | 851.82 | 0.0031 | 0.23 |
| P27918 | Properdin (*CFP*) | 6 | 0.282 | 186.73 | 0.014 | 0.58 |
| P05160 | Coagulation factor XIII B chain (*F13B*) | 8 | 0.211 | 394.33 | 0.029 | 0.61 |

**S1 Table 5**. Regression analysis of survival time versus log fold change in cytokine densities across time points. ***β*** is the regression coefficient, the p-values are for a t-test of the difference of ***β*** from 0. Because of the small number of associations, the time point intervals are combined in single tables. Sample sizes for A/B, A/C, and B/C comparisons are, respectively N = 13, 13, 9.

**S1 Table 5a:** Progression-free survival time (PFS)

| **Cytokine ID** | **Protein (Gene Name)** | **Time Points** | **Log FC** | ***β*** | ***p*** | ***p**** |
| --- | --- | --- | --- | --- | --- | --- |
| MIG | Chemokine ligand 9 (*CXCL9*) | B/A | -1.18 | -98.19 | 0.017 | 0.53 |
| HGF | Hepatocyte growth factor | C/A | 0.257 | -126.96 | 0.045 | 0.60 |
| TNFa | Tumor necrosis factor - alpha | C/A | -0.095 | -123.30 | 0.046 | 1 |

**S1 Table 5b:** Regression analysis of changes in cytokine concentration vs. right-censored overall survival time (OS). N = 9, 9, 6 for B/A, C/A, C/B.

| **Cytokine ID** | **Protein (Gene Name)** | **Time Points** | **Log FC** | ***β*** | ***p*** | ***p**** |
| --- | --- | --- | --- | --- | --- | --- |
| Angiopoietin 2 | (*ANGPT2*) | B/A | 0.022 | -198.13 | 0.031 | 0.25 |
| Eotaxin | C-C motif chemokine 14 (*CCL14*) | B/A | 0.122 | 310.00 | 0.024 | 0.25 |
| OPN | Osteopontin | B/A | 0.038 | -156.24 | 0.037 | 0.25 |
| MIG | Chemokine ligand 9 (*CXCL9*) | C/A | -0.769 | -310.98 | 0.012 | 0.37 |
| SCF | Stem Cell Factor | C/A | -0.485 | 111.02 | 0.019 | 0.29 |
| ICAM 1 | Intercellular Adhesion Molecular 1 | C/B | 0.614 | -539.50 | 0.038 | 0.51 |

**S1 Table 6**. Changes in protein density (log FC) across time points in relation to mean dosage (Gy) to tumor. ***β*** is the regression coefficient, the p-values are for a t-test of the difference of ***β*** from 0. p-values are adjusted via Benjamini-Hochberg FDR. Tumor dosage varies due to differences in missing sample points.

**S1 Table 6a:** B vs. A

| **Protein Accession** | **Protein (Gene Name)** | **Mean Log FC (B/A)** | **Mean tumor dose (Gy)** | ***β*** | ***p*** | ***p**** |
| --- | --- | --- | --- | --- | --- | --- |
| P04278 | Sex hormone binding globulin (*SHBG*) | -0.340 | 40.02 | -0.0619 | 0.0062 | 0.32 |
| P07225 | Vitamin K dependent protein S (*VKDPS*) | 0.033 | 37.67 | 0.0317 | 0.0035 | 0.27 |
| P33151 | Cadherin-5  (*CDH5*) | -0.529 | 42.57 | 0.0206 | 0.0021 | 0.27 |
| Q92954 | Proteoglycan 4  (*PRG4*) | 0.305 | 34.20 | 0.0310 | 0.031 | 0.59 |
| P61769 | Beta-2-microglobulin (*B2M*) | -0.229 | 36.49 | -0.0363 | 0.031 | 0.59 |
| P69905 | Hemoglobin subunit alpha (*HBA1*) | 0.680 | 36.86 | 0.0380 | 0.018 | 0.55 |
| Q93073 | Selenocysteine insertion sequence binding protein 2-like (*SECISBP2L*) | 0.915 | 28.37 | -0.0495 | 0.014 | 0.55 |
| A0A087WVQ6 | Clathrin heavy chain (*CLTC*) | 0.147 | 28.37 | 0.0129 | 0.023 | 0.59 |

**S1 Table 6b:** C/A

| **Protein Accession** | **Protein (Gene Name)** | **Mean Log FC protein (log C/A)** | **Mean tumor dose (Gy)** | ***β*** | ***p*** | ***p**** |
| --- | --- | --- | --- | --- | --- | --- |
| P10909-2 | Isoform 2 of Clusterin (*CLU*) | -0.081 | 32.26 | -0.0093 | 0.018 | 0.98 |
| P07358 | Complement component C8 beta chain (*C8B*) | 0.0869 | 32.26 | 0.0105 | 0.049 | 0.98 |
| P01023 | Alpha-2-macroglobulin (*A2M*) | 0.0862 | 32.26 | 0.0368 | 0.040 | 0.98 |
| O75882 | Attractin (*ATRN*) | 0.181 | 32.26 | 0.0246 | 0.045 | 0.98 |
| A0A140TA32 | Complement C4-A | -0.085 | 31.74 | 0.00584 | 0.019 | 0.98 |
| A0A140TA29 | Complement C4-B | -0.082 | 31.74 | 0.00566 | 0.031 | 0.98 |
| P00742 | Coagulation factor X (*F10*) | 0.088 | 32.26 | 0.0250 | 0.015 | 0.98 |
| P02787 | Serotranferrin (*TF*) | -0.788 | 32.26 | 0.0511 | 0.044 | 0.98 |
| A0A087WT59 | Transthyretin (*TTR*) | -0.208 | 32.10 | 0.0549 | 0.0064 | 0.98 |
| Q93073 | Selenocysteine insertion sequence-binding protein 2-like (*SECISBP2L*) | -0.123 | 28.37 | -0.0616 | 0.0742 | 0.98 |

**S1 Table 6c:** B/C

| **Protein Accession** | **Protein (Gene Name)** | **Mean Log FC SAF (B/A)** | **Mean tumor dose (Gy)** | ***β*** | ***p*** | ***p**** |
| --- | --- | --- | --- | --- | --- | --- |
| G3V2W1 | Protein Z-dependent protease inhibitor (*SERPINA10*) | -1.012 | 34.20 | -0.0356 | 0.048 | 0.94 |
| G6UY11 | Protein delta homolog 2 (*DLK2*) | 0.693 | 32.93 | 0.0527 | 0.010 | 0.96 |
| A0A075B6Z2 | T cell receptor alpha joinint 46 (*TRAJ56*) | -1.25 | 34.20 | -0.0278 | 0.030 | 0.94 |
| E7ETH0 | Complement Factor I (*CFI*) | -0.088 | 34.20 | -0.0074 | 0.016 | 0.94 |
| P02760 | Alpha macroglobulin precursor (*AMBP*) | 0 | 34.20 | 0.0006 | 0.038 | 0.82 |
| P15169 | Carboxypeptidase N (*CPA-N*) | -0.208 | 34.20 | -0.0137 | 0.010 | 0.94 |

**S1 Table 7**. Regression of changes in cytokine concentrations across time points against mean dosage (Gy) to the tumor. ***β*** is the regression coefficient, the p-values are for a t-test of the difference of ***β*** from 0. Note: mean tumor dose is not the same for all cytokine data within a time interval comparison because of missing individual data and different sample sizes

| **Cytokine ID** | **Cytokine name** | **Time Point Comparison**  **and Sample Size** | **Mean Log FC** | **Mean tumor dose (Gy)** | ***β*** | ***p*** | ***p**** |
| --- | --- | --- | --- | --- | --- | --- | --- |
| HGF | Heptocylte growth factor (*HGF*) | B/A, N = 8 | 0.597 | 37.35 | 0.053 | 0.032 | 0.50 |
| MMP | Matrix metalloprotease (*MMP*) | B/A, N = 4 | 0.320 | 42.17 | 0.095 | 0.004 | 0.11 |
| EGF | Epidermal growth factor (*EGF*) | C/A, N = 5 | 0.296 | 23.96 | 0.146 | 0.028 | 0.44 |
| MIG | Chemokine ligand 9 (*CXCL9*) | C/A, N = 12  C/B, N = 12 | -0.786  0.696 | 26.03  25.78 | 0.036  0.028 | 0.019  0.017 | 0.44  0.27 |
| HIGF1 | Human insulin-like growth factor (*HIGF1*) | C/B, N = 6 | -0.254 | 29.74 | 0.047 | 0.017 | 0.27 |

**S1 Table 8**. Regression analysis of blood chemistry measures vs. mean radiation dosage to tumor. ***β*** is the regression coefficient, the p-values are for a t-test of the difference of ***β*** from 0.

Change = Last Treatment – First Screening

Difference = Mean Treatment – Mean Screening

| ***Blood Chemistry*** | ***Model*** | ***Mean at Screening*** | ***Summary Statistic*** | ***β*** | ***p*** | ***p**** |
| --- | --- | --- | --- | --- | --- | --- |
| CEA | Change vs. Dosage Ratio | 35.54 (ng/mL) | Change = 15.67 | -202.15 | 0.046 | 0.61 |
| Alkaline Phosphatase | Difference vs. Dosage | 158.21 (U/L) | Difference = 22.35 | -3.02 | 0.012 | 0.065 |
| SGOT/AST | Difference vs. Dosage Ratio | 41.5 (U/L) | Difference = 5.25 | -0.748 | 0.018 | 0.30 |
| *Total Bilirubin* | *Difference vs. Dosage* | *0.6 (mg/dL)* | *Difference = 0.18* | *-0.01* | *0.006* | *0.048* |
| Potassium | Difference vs. Dosage | 4.62 (mmol/L) | Difference = -0.37 | -0.028 | 0.039 | 0.17 |

Marginal significance: SGPT/ALT vs. dosage ratio (mean at screening = 53.79 U/L mean difference = -2.22 coefficient = 65.69, p = 0.051, adjusted p* = 0.43)
