# Supplementary material for: Regorafenib Prior to Selective Internal Radiation Therapy Using 90Y-Resin Microspheres for Refractory Metastatic Colorectal Cancer Liver Metastases: Analysis of Safety, Dosimetry, and Molecular Markers
Source: Front Oncol. 2019 Jul 10;9:624. doi: 10.3389/fonc.2019.00624 (PMC6636394; doi:10.3389/fonc.2019.00624)
Supplement: Supplementary file 2 [file Data_Sheet_2.docx]

**Supplementary Data S2**

Enrichment analysis (clustering and annotation using DAVID) of proteins with significant FC in density across pre- and post-treatment time points.

***Differentially Expressed Genes***

(identified on the basis of ms/ms SAF log FC p < 0.05)

*CP, TF, CLEC3B, CFP, BCHE, DLK2, TRAJ56, KLKB1, SERPINF2, AGT, RBP4, F9, SHBG, C4A, VWF, C8B, HRG, CFHR5, B2M*

***S2 Table 1: Functional Annotation Chart***

Category Term Count % PValue Genes List Total Pop Hits Pop Total Fold Enrichment Bonferroni Benjamini FDR

GOTERM_CC_DIRECT GO:0005576~extracellular region 17 0.6060606060606061 1.9950875388894295E-16 SHBG, RBP4, TF, C4A, F9, B2M, CFP, VWF, C8B, SERPINF2, BCHE, CLEC3B, AGT, KLKB1, HRG, CP, CFHR5 18 1610 18224 10.6904071773637 1.3322676295501878E-14 1.3322676295501878E-14 2.220446049250313E-13

UP_KEYWORDS Secreted 17 0.6060606060606061 5.766676872319847E-15 SHBG, RBP4, TF, C4A, F9, B2M, CFP, VWF, C8B, SERPINF2, BCHE, CLEC3B, AGT, KLKB1, HRG, CP, CFHR5 19 1965 20581 9.371286996116245 4.791722574282176E-13 4.791722574282176E-13 6.183942247162122E-12

UP_SEQ_FEATURE signal peptide 18 0.6417112299465241 5.773970907488748E-14 SHBG, RBP4, TF, C4A, F9, DLK2, B2M, CFP, VWF, C8B, SERPINF2, BCHE, CLEC3B, AGT, KLKB1, HRG, CP, CFHR5 18 3346 20063 5.99611476389719 8.37108160567368E-12 8.37108160567368E-12 6.853406731011091E-11

UP_SEQ_FEATURE disulfide bond 17 0.6060606060606061 5.651949147220255E-13 SHBG, RBP4, TF, C4A, F9, DLK2, B2M, CFP, VWF, C8B, CLEC3B, SERPINF2, BCHE, KLKB1, HRG, CP, CFHR5 18 2917 20063 6.495848093551213 8.195610856631674E-11 4.09781097943096E-11 6.709965916229521E-10

UP_KEYWORDS Disulfide bond 18 0.6417112299465241 8.839804687690061E-13 SHBG, RBP4, TF, C4A, F9, DLK2, B2M, CFP, VWF, C8B, SERPINF2, BCHE, CLEC3B, AGT, KLKB1, HRG, CP, CFHR5 19 3434 20581 5.677865309750789 7.336864449314362E-11 3.668432224657181E-11 9.46598355255901E-10

UP_KEYWORDS Signal 18 0.6417112299465241 2.2280239431667174E-11 SHBG, RBP4, TF, C4A, F9, DLK2, B2M, CFP, VWF, C8B, SERPINF2, BCHE, CLEC3B, AGT, KLKB1, HRG, CP, CFHR5 19 4160 20581 4.686968623481781 1.8492639641820574E-9 6.164213584014533E-10 2.385920350178594E-8

GOTERM_CC_DIRECT GO:0070062~extracellular exosome 15 0.53475935828877 1.795512002092053E-9 SHBG, RBP4, TF, C4A, F9, DLK2, B2M, VWF, C8B, SERPINF2, CLEC3B, AGT, KLKB1, HRG, CP 18 2811 18224 5.402585082414325 1.0773071201786166E-7 5.386535739670961E-8 1.7994012835309547E-6

GOTERM_CC_DIRECT GO:0005615~extracellular space 12 0.42780748663101603 2.8242733616227057E-9 CFP, TF, C8B, RBP4, C4A, CLEC3B, SERPINF2, AGT, KLKB1, F9, CP, B2M 18 1347 18224 9.019549616431576 1.6945638814380715E-7 5.6485465860234285E-8 2.8303911103755297E-6

UP_KEYWORDS Glycoprotein 17 0.6060606060606061 3.0764460301393948E-9 SHBG, TF, C4A, F9, DLK2, B2M, CFP, VWF, C8B, SERPINF2, BCHE, CLEC3B, AGT, KLKB1, HRG, CP, CFHR5 19 4551 20581 4.046270917901213 2.553449847120248E-7 6.383625228423284E-8 3.2944617256447373E-6

GOTERM_CC_DIRECT GO:0072562~blood microparticle 7 0.24955436720142604 3.49816007883126E-9 TF, C4A, BCHE, SERPINF2, AGT, HRG, CP 18 152 18224 46.62573099415204 2.0988958404632996E-7 5.2472400091652105E-8 3.505737555808963E-6

KEGG_PATHWAY hsa04610:Complement and coagulation cascades 6 0.21390374331550802 2.1251296338160597E-8 C8B, VWF, C4A, SERPINF2, KLKB1, F9 11 69 6879 54.3794466403162 3.61271977222799E-7 3.61271977222799E-7 1.5387030083413578E-5

UP_KEYWORDS Disease mutation 13 0.4634581105169341 1.1513582629941925E-7 RBP4, TF, C4A, F9, B2M, CFP, VWF, SERPINF2, BCHE, AGT, KLKB1, HRG, CFHR5 19 2550 20581 5.522249742002065 9.556228473428519E-6 1.9112530004194994E-6 1.2329498670915484E-4

UP_SEQ_FEATURE glycosylation site:N-linked (GlcNAc...) 15 0.53475935828877 1.2063581808053843E-7 SHBG, TF, C4A, F9, DLK2, CFP, VWF, C8B, SERPINF2, BCHE, AGT, KLKB1, HRG, CP, CFHR5 18 4234 20063 3.9487875925051172 1.7492041693878413E-5 5.83071456183859E-6 1.4321385660709396E-4

UP_SEQ_FEATURE glycosylation site:O-linked (GalNAc...) 5 0.17825311942959002 1.1642668677911453E-6 SHBG, TF, VWF, CLEC3B, F9 18 97 20063 57.45418098510882 1.6880454501577713E-4 4.220380792085976E-5 0.0013821616577702756

GOTERM_BP_DIRECT GO:0002576~platelet degranulation 5 0.17825311942959002 2.295892004498356E-6 TF, VWF, CLEC3B, SERPINF2, HRG 17 103 16792 47.94974300399772 5.302110502737323E-4 5.302110502737323E-4 0.002945428120681992

UP_KEYWORDS Hemostasis 4 0.14260249554367202 9.479015074902825E-6 VWF, KLKB1, F9, HRG 19 48 20581 90.26754385964912 7.864525640282061E-4 1.31118399911756E-4 0.010150289028898474

UP_KEYWORDS Blood coagulation 4 0.14260249554367202 9.479015074902825E-6 VWF, KLKB1, F9, HRG 19 48 20581 90.26754385964912 7.864525640282061E-4 1.31118399911756E-4 0.010150289028898474

GOTERM_BP_DIRECT GO:0006957~complement activation, alternative pathway 3 0.10695187165775401 6.598889367680451E-5 CFP, C8B, CFHR5 17 13 16792 227.94570135746608 0.015128336744814175 0.007592995160158211 0.08462607911664533

GOTERM_BP_DIRECT GO:0007597~blood coagulation, intrinsic pathway 3 0.10695187165775401 1.2908050027524043E-4 VWF, KLKB1, F9 17 18 16792 164.62745098039218 0.029379304794927785 0.009890603115410568 0.16547483987693345

GOTERM_BP_DIRECT GO:0042730~fibrinolysis 3 0.10695187165775401 1.768741457509123E-4 SERPINF2, KLKB1, HRG 17 21 16792 141.109243697479 0.04003796430198714 0.01016338553021856 0.22667986324464984

GOTERM_BP_DIRECT GO:0010951~negative regulation of endopeptidase activity 4 0.14260249554367202 1.908479532798974E-4 C4A, SERPINF2, AGT, HRG 17 121 16792 32.65337870685464 0.04313224545161609 0.008779252233582557 0.24456832405580498

UP_SEQ_FEATURE sequence variant 18 0.6417112299465241 2.95932554651353E-4 SHBG, RBP4, TF, C4A, F9, DLK2, B2M, CFP, VWF, C8B, SERPINF2, BCHE, CLEC3B, AGT, KLKB1, HRG, CP, CFHR5 18 12443 20063 1.6123925098448926 0.04200868895512144 0.008546582715076134 0.3507545341466911

GOTERM_BP_DIRECT GO:0030449~regulation of complement activation 3 0.10695187165775401 3.645542798934609E-4 CFP, C8B, C4A 17 30 16792 98.7764705882353 0.08077779173927335 0.013939826863130889 0.46669039681971336

GOTERM_CC_DIRECT GO:0005788~endoplasmic reticulum lumen 4 0.14260249554367202 7.021048449333655E-4 CFP, BCHE, F9, B2M 18 192 18224 21.09259259259259 0.04126549313814254 0.008392799381729432 0.701401446948291

UP_KEYWORDS Sulfation 3 0.10695187165775401 8.98037047783165E-4 C4A, SERPINF2, F9 19 51 20581 63.71826625386996 0.07185802123932172 0.010596396064996072 0.9574960525179144

GOTERM_CC_DIRECT GO:0031093~platelet alpha granule lumen 3 0.10695187165775401 0.001181423677255857 VWF, SERPINF2, HRG 18 55 18224 55.22424242424242 0.06847041281817856 0.01175162495663229 1.177693017501702

UP_KEYWORDS Cleavage on pair of basic residues 4 0.14260249554367202 0.0020082847400068595 VWF, C4A, F9, HRG 19 294 20581 14.737558181167204 0.15367788052903553 0.020640903573544755 2.1297598667544437

UP_SEQ_FEATURE glycosylation site:O-linked (Fuc...) 2 0.07130124777183601 0.002539965918699014 CFP, F9 18 3 20063 743.074074074074 0.30841110355416834 0.059610000518299566 2.9740584477175758

GOTERM_MF_DIRECT GO:0019865~immunoglobulin binding 2 0.07130124777183601 0.002663508519138912 VWF, HRG 16 3 16881 703.375 0.1433189450229394 0.1433189450229394 2.6186547815059136

GOTERM_BP_DIRECT GO:0002034~regulation of blood vessel size by renin-angiotensin 2 0.07130124777183601 0.002855951155586763 SERPINF2, AGT 17 3 16792 658.5098039215686 0.4834941091934355 0.09006419549889055 3.602726425423497

GOTERM_BP_DIRECT GO:0006956~complement activation 3 0.10695187165775401 0.003037495016610305 CFP, C8B, C4A 17 87 16792 34.06085192697769 0.5047681083451294 0.08409363831225836 3.8276441472065548

GOTERM_MF_DIRECT GO:0004867~serine-type endopeptidase inhibitor activity 3 0.10695187165775401 0.0032680761460585064 SERPINF2, AGT, HRG 16 97 16881 32.63079896907217 0.1729241096031321 0.09056287166353938 3.2043824628469886

GOTERM_BP_DIRECT GO:0071281~cellular response to iron ion 2 0.07130124777183601 0.005704254266997648 TF, B2M 17 6 16792 329.2549019607843 0.733253173985301 0.13655783865464255 7.076266055349523

UP_KEYWORDS Fibrinolysis 2 0.07130124777183601 0.006976547313657517 KLKB1, HRG 19 8 20581 270.80263157894734 0.4407075453147158 0.06252459109904163 7.222989365850285

GOTERM_CC_DIRECT GO:1990712~HFE-transferrin receptor complex 2 0.07130124777183601 0.0074397911556696285 TF, B2M 18 8 18224 253.1111111111111 0.3611312637375478 0.06200254015877704 7.210602462581061

GOTERM_BP_DIRECT GO:0031638~zymogen activation 2 0.07130124777183601 0.008544928453937934 KLKB1, F9 17 9 16792 219.50326797385623 0.8622562424842796 0.17982374356251107 10.425229412936165

UP_KEYWORDS Thrombophilia 2 0.07130124777183601 0.008713487647492343 F9, HRG 19 10 20581 216.64210526315787 0.516347584776751 0.07006341009981243 8.946113099382647

UP_KEYWORDS Immunity 4 0.14260249554367202 0.008863209541506902 CFP, C8B, C4A, B2M 19 500 20581 8.665684210526315 0.5223733307073185 0.06496851815662419 9.09327703269044

GOTERM_BP_DIRECT GO:0051918~negative regulation of fibrinolysis 2 0.07130124777183601 0.009490127830826386 SERPINF2, HRG 17 10 16792 197.55294117647057 0.8894942844567182 0.18146929310691162 11.514640096470108

UP_KEYWORDS Complement alternate pathway 2 0.07130124777183601 0.010447558330780076 CFP, C8B 19 12 20581 180.53508771929825 0.5817637970005529 0.07006669512285713 10.637396651779818

UP_SEQ_FEATURE glycosylation site:C-linked (Man) 2 0.07130124777183601 0.011801321223155348 CFP, C8B 18 14 20063 159.23015873015873 0.8211788058609826 0.21800727466154268 13.145314618289882

UP_SEQ_FEATURE active site:Charge relay system 3 0.10695187165775401 0.012537309845243526 BCHE, KLKB1, F9 18 203 20063 16.47208538587849 0.8394904651716316 0.20441313476302625 13.910156497444582

GOTERM_BP_DIRECT GO:0048260~positive regulation of receptor-mediated endocytosis 2 0.07130124777183601 0.017958989194505382 TF, B2M 17 19 16792 103.97523219814242 0.98479650999294 0.294502832546619 20.744645216561388

UP_KEYWORDS EGF-like domain 3 0.10695187165775401 0.01803643326299921 C8B, F9, DLK2 19 238 20581 13.65391419725785 0.779242178594145 0.10970894965029199 17.709055753144774

GOTERM_MF_DIRECT GO:0004252~serine-type endopeptidase activity 3 0.10695187165775401 0.020965802900630586 C4A, KLKB1, F9 16 255 16881 12.4125 0.7073988828204352 0.3361163190191858 19.007510882150967

UP_KEYWORDS Innate immunity 3 0.10695187165775401 0.02144367197635731 CFP, C8B, C4A 19 261 20581 12.45069570477919 0.8345663438481173 0.12059806044419352 20.71576499658161

UP_KEYWORDS Polymorphism 16 0.5704099821746881 0.024627105487666338 SHBG, TF, C4A, F9, DLK2, CFP, VWF, C8B, SERPINF2, BCHE, CLEC3B, AGT, KLKB1, HRG, CP, CFHR5 19 12043 20581 1.4391238413229785 0.8737688199590771 0.1288804185298733 23.434608565355074

UP_KEYWORDS Complement pathway 2 0.07130124777183601 0.026778956588305997 C8B, C4A 19 31 20581 69.88455008488964 0.8949118511625989 0.13134542991204656 25.224237616748457

GOTERM_BP_DIRECT GO:0055072~iron ion homeostasis 2 0.07130124777183601 0.028217634556604167 TF, B2M 17 30 16792 65.85098039215686 0.9986558671546568 0.3986726765999451 30.734147749508235

INTERPRO IPR023795:Protease inhibitor I4, serpin, conserved site 2 0.07130124777183601 0.029814403247814373 SERPINF2, AGT 18 33 18559 62.48821548821549 0.9213307300089132 0.9213307300089132 27.739386467963023

GOTERM_CC_DIRECT GO:0031012~extracellular matrix 3 0.10695187165775401 0.03045083706214646 CFP, VWF, CLEC3B 18 296 18224 10.26126126126126 0.8436167822949262 0.20699893951306203 26.648763228807248

INTERPRO IPR023796:Serpin domain 2 0.07130124777183601 0.03425807376783304 SERPINF2, AGT 18 38 18559 54.26608187134502 0.9465027782346168 0.7687053356314003 31.21382290169974

INTERPRO IPR000215:Serpin family 2 0.07130124777183601 0.03425807376783304 SERPINF2, AGT 18 38 18559 54.26608187134502 0.9465027782346168 0.7687053356314003 31.21382290169974

GOTERM_MF_DIRECT GO:0004866~endopeptidase inhibitor activity 2 0.07130124777183601 0.03497365418308537 C4A, SERPINF2 16 40 16881 52.753125 0.8731554357649843 0.403214908682901 29.826221853272983

GOTERM_BP_DIRECT GO:0001895~retina homeostasis 2 0.07130124777183601 0.037456412777707754 TF, B2M 17 40 16792 49.38823529411764 0.9998520504861413 0.4673564386351702 38.72328919357875

UP_SEQ_FEATURE domain:TSP type-1 1 2 0.07130124777183601 0.03746799645033003 CFP, C8B 18 45 20063 49.538271604938274 0.9960626785626615 0.4594945257870888 36.450560463698714

UP_SEQ_FEATURE domain:TSP type-1 2 2 0.07130124777183601 0.03746799645033003 CFP, C8B 18 45 20063 49.538271604938274 0.9960626785626615 0.4594945257870888 36.450560463698714

GOTERM_BP_DIRECT GO:0006879~cellular iron ion homeostasis 2 0.07130124777183601 0.04112881551346262 TF, CP 17 44 16792 44.898395721925134 0.9999388182603833 0.47626919225959996 41.65588588266307

SMART SM00093:SERPIN 2 0.07130124777183601 0.05032440875754256 SERPINF2, AGT 15 37 10057 36.24144144144144 0.7644366494037936 0.7644366494037936 35.07635214268912

UP_KEYWORDS Copper 2 0.07130124777183601 0.05537010763061479 HRG, CP 19 65 20581 33.329554655870446 0.9911545524070415 0.24278569032532782 45.66423816142887

GOTERM_MF_DIRECT GO:0001948~glycoprotein binding 2 0.07130124777183601 0.05624887011951108 VWF, B2M 16 65 16881 32.46346153846154 0.9651875887031972 0.4890871554138496 43.785490525223366

GOTERM_BP_DIRECT GO:0016525~negative regulation of angiogenesis 2 0.07130124777183601 0.05749266773783254 AGT, HRG 17 62 16792 31.863377609108156 0.9999988524427481 0.5746603223359307 53.21665708029235

INTERPRO IPR000884:Thrombospondin, type 1 repeat 2 0.07130124777183601 0.057924750694272206 CFP, C8B 18 65 18559 31.724786324786326 0.9933443589713312 0.8118966067007582 47.29659665149653

GOTERM_BP_DIRECT GO:0006955~immune response 3 0.10695187165775401 0.059694207041121564 CFP, C8B, B2M 17 421 16792 7.0387033673326815 0.9999993314024225 0.5667142561567464 54.599411155163025

GOTERM_MF_DIRECT GO:0051087~chaperone binding 2 0.07130124777183601 0.06963439573207295 VWF, CP 16 81 16881 26.050925925925927 0.9847976238650786 0.5022798244900619 51.233196235588686

UP_SEQ_FEATURE domain:EGF-like 2 2 0.07130124777183601 0.07282266680373962 F9, DLK2 18 89 20063 25.04744069912609 0.9999826776484962 0.6659121476464367 59.24600863823115

GOTERM_BP_DIRECT GO:0006508~proteolysis 3 0.10695187165775401 0.08065829881722537 C4A, KLKB1, F9 17 500 16792 5.926588235294118 0.9999999963425552 0.66014970248586 66.00343320073763

BIOCARTA h_intrinsicPathway:Intrinsic Prothrombin Activation Pathway 2 0.07130124777183601 0.0820961490824833 KLKB1, F9 7 23 1625 20.186335403726705 0.7233326705179974 0.7233326705179974 44.87558739930949

GOTERM_MF_DIRECT GO:0002020~protease binding 2 0.07130124777183601 0.08611714341717755 VWF, SERPINF2 16 101 16881 20.892326732673265 0.9946092304012837 0.5258124905415584 59.17874309137501

SMART SM00209:TSP1 2 0.07130124777183601 0.08683329773928854 CFP, C8B 15 65 10057 20.62974358974359 0.9214037409455156 0.7196497564572408 53.229048149162914

UP_SEQ_FEATURE domain:Peptidase S1 2 0.07130124777183601 0.08848562900674323 KLKB1, F9 18 109 20063 20.451580020387357 0.9999985354290247 0.7051439532642693 66.70884747905941

INTERPRO IPR018097:EGF-like calcium-binding, conserved site 2 0.07130124777183601 0.08947140432308888 F9, DLK2 18 102 18559 20.21677559912854 0.9996192310489315 0.8603099792371756 63.43517407639332

INTERPRO IPR018114:Peptidase S1, trypsin family, active site 2 0.07130124777183601 0.08947140432308888 KLKB1, F9 18 102 18559 20.21677559912854 0.9996192310489315 0.8603099792371756 63.43517407639332

GOTERM_BP_DIRECT GO:0006958~complement activation, classical pathway 2 0.07130124777183601 0.0903107400015329 C8B, C4A 17 99 16792 19.954842543077838 0.9999999996806267 0.6836074563640211 70.30885550444127

INTERPRO IPR000152:EGF-type aspartate/asparagine hydroxylation site 2 0.07130124777183601 0.09198517751774847 F9, DLK2 18 105 18559 19.639153439153436 0.9996981391475759 0.8023206252143049 64.5042876307344

INTERPRO IPR001314:Peptidase S1A, chymotrypsin-type 2 0.07130124777183601 0.09865667772410647 KLKB1, F9 18 113 18559 18.248770894788592 0.9998375272144792 0.7664050564418904 67.20567646183389

***S2 Table 2: Functional Annotation Clustering***

Annotation Cluster 1 Enrichment Score: 11.271479377854948

Category Term Count % PValue Genes List Total Pop Hits Pop Total Fold Enrichment Bonferroni Benjamini FDR

GOTERM_CC_DIRECT GO:0005576~extracellular region 17 0.6060606060606061 1.9950875388894295E-16 SHBG, RBP4, TF, C4A, F9, B2M, CFP, VWF, C8B, SERPINF2, BCHE, CLEC3B, AGT, KLKB1, HRG, CP, CFHR5 18 1610 18224 10.6904071773637 1.3322676295501878E-14 1.3322676295501878E-14 2.220446049250313E-13

UP_KEYWORDS Secreted 17 0.6060606060606061 5.766676872319847E-15 SHBG, RBP4, TF, C4A, F9, B2M, CFP, VWF, C8B, SERPINF2, BCHE, CLEC3B, AGT, KLKB1, HRG, CP, CFHR5 19 1965 20581 9.371286996116245 4.791722574282176E-13 4.791722574282176E-13 6.183942247162122E-12

UP_SEQ_FEATURE signal peptide 18 0.6417112299465241 5.773970907488748E-14 SHBG, RBP4, TF, C4A, F9, DLK2, B2M, CFP, VWF, C8B, SERPINF2, BCHE, CLEC3B, AGT, KLKB1, HRG, CP, CFHR5 18 3346 20063 5.99611476389719 8.37108160567368E-12 8.37108160567368E-12 6.853406731011091E-11

UP_SEQ_FEATURE disulfide bond 17 0.6060606060606061 5.651949147220255E-13 SHBG, RBP4, TF, C4A, F9, DLK2, B2M, CFP, VWF, C8B, CLEC3B, SERPINF2, BCHE, KLKB1, HRG, CP, CFHR5 18 2917 20063 6.495848093551213 8.195610856631674E-11 4.09781097943096E-11 6.709965916229521E-10

UP_KEYWORDS Disulfide bond 18 0.6417112299465241 8.839804687690061E-13 SHBG, RBP4, TF, C4A, F9, DLK2, B2M, CFP, VWF, C8B, SERPINF2, BCHE, CLEC3B, AGT, KLKB1, HRG, CP, CFHR5 19 3434 20581 5.677865309750789 7.336864449314362E-11 3.668432224657181E-11 9.46598355255901E-10

UP_KEYWORDS Signal 18 0.6417112299465241 2.2280239431667174E-11 SHBG, RBP4, TF, C4A, F9, DLK2, B2M, CFP, VWF, C8B, SERPINF2, BCHE, CLEC3B, AGT, KLKB1, HRG, CP, CFHR5 19 4160 20581 4.686968623481781 1.8492639641820574E-9 6.164213584014533E-10 2.385920350178594E-8

UP_KEYWORDS Glycoprotein 17 0.6060606060606061 3.0764460301393948E-9 SHBG, TF, C4A, F9, DLK2, B2M, CFP, VWF, C8B, SERPINF2, BCHE, CLEC3B, AGT, KLKB1, HRG, CP, CFHR5 19 4551 20581 4.046270917901213 2.553449847120248E-7 6.383625228423284E-8 3.2944617256447373E-6

UP_SEQ_FEATURE sequence variant 18 0.6417112299465241 2.95932554651353E-4 SHBG, RBP4, TF, C4A, F9, DLK2, B2M, CFP, VWF, C8B, SERPINF2, BCHE, CLEC3B, AGT, KLKB1, HRG, CP, CFHR5 18 12443 20063 1.6123925098448926 0.04200868895512144 0.008546582715076134 0.3507545341466911

Annotation Cluster 2 Enrichment Score: 5.679687018324865

Category Term Count % PValue Genes List Total Pop Hits Pop Total Fold Enrichment Bonferroni Benjamini FDR

UP_KEYWORDS Glycoprotein 17 0.6060606060606061 3.0764460301393948E-9 SHBG, TF, C4A, F9, DLK2, B2M, CFP, VWF, C8B, SERPINF2, BCHE, CLEC3B, AGT, KLKB1, HRG, CP, CFHR5 19 4551 20581 4.046270917901213 2.553449847120248E-7 6.383625228423284E-8 3.2944617256447373E-6

UP_SEQ_FEATURE glycosylation site:N-linked (GlcNAc...) 15 0.53475935828877 1.2063581808053843E-7 SHBG, TF, C4A, F9, DLK2, CFP, VWF, C8B, SERPINF2, BCHE, AGT, KLKB1, HRG, CP, CFHR5 18 4234 20063 3.9487875925051172 1.7492041693878413E-5 5.83071456183859E-6 1.4321385660709396E-4

UP_KEYWORDS Polymorphism 16 0.5704099821746881 0.024627105487666338 SHBG, TF, C4A, F9, DLK2, CFP, VWF, C8B, SERPINF2, BCHE, CLEC3B, AGT, KLKB1, HRG, CP, CFHR5 19 12043 20581 1.4391238413229785 0.8737688199590771 0.15841648446374146 23.434608565355074

Annotation Cluster 3 Enrichment Score: 3.5747482030651723

Category Term Count % PValue Genes List Total Pop Hits Pop Total Fold Enrichment Bonferroni Benjamini FDR

KEGG_PATHWAY hsa04610:Complement and coagulation cascades 6 0.21390374331550802 2.1251296338160597E-8 C8B, VWF, C4A, SERPINF2, KLKB1, F9 11 69 6879 54.3794466403162 3.61271977222799E-7 3.61271977222799E-7 1.5387030083413578E-5

UP_KEYWORDS Sulfation 3 0.10695187165775401 8.98037047783165E-4 C4A, SERPINF2, F9 19 51 20581 63.71826625386996 0.07185802123932172 0.010596396064996072 0.9574960525179144

UP_KEYWORDS Alternative splicing 6 0.21390374331550802 0.9887254427831726 SHBG, VWF, C4A, SERPINF2, F9, DLK2 19 10587 20581 0.6138909188528134 1.0 0.9999999996912107 100.0

Annotation Cluster 4 Enrichment Score: 2.8812026979305263

Category Term Count % PValue Genes List Total Pop Hits Pop Total Fold Enrichment Bonferroni Benjamini FDR

UP_KEYWORDS Hemostasis 4 0.14260249554367202 9.479015074902825E-6 VWF, KLKB1, F9, HRG 19 48 20581 90.26754385964912 7.864525640282061E-4 1.31118399911756E-4 0.010150289028898474

UP_KEYWORDS Blood coagulation 4 0.14260249554367202 9.479015074902825E-6 VWF, KLKB1, F9, HRG 19 48 20581 90.26754385964912 7.864525640282061E-4 1.31118399911756E-4 0.010150289028898474

GOTERM_BP_DIRECT GO:0007597~blood coagulation, intrinsic pathway 3 0.10695187165775401 1.2908050027524043E-4 VWF, KLKB1, F9 17 18 16792 164.62745098039218 0.029379304794927785 0.009890603115410568 0.16547483987693345

UP_KEYWORDS Cleavage on pair of basic residues 4 0.14260249554367202 0.0020082847400068595 VWF, C4A, F9, HRG 19 294 20581 14.737558181167204 0.15367788052903553 0.020640903573544755 2.1297598667544437

UP_KEYWORDS Metal-binding 5 0.17825311942959002 0.3973246308976204 TF, KLKB1, F9, HRG, CP 19 3640 20581 1.4879265471370733 1.0 0.9605617746816437 99.5584416583584

GOTERM_CC_DIRECT GO:0005886~plasma membrane 5 0.17825311942959002 0.5577325197977883 C4A, KLKB1, F9, HRG, B2M 18 4121 18224 1.2283965596268436 1.0 0.9977989112580559 99.97186965236665

Annotation Cluster 5 Enrichment Score: 2.6745070120766568

Category Term Count % PValue Genes List Total Pop Hits Pop Total Fold Enrichment Bonferroni Benjamini FDR

KEGG_PATHWAY hsa04610:Complement and coagulation cascades 6 0.21390374331550802 2.1251296338160597E-8 C8B, VWF, C4A, SERPINF2, KLKB1, F9 11 69 6879 54.3794466403162 3.61271977222799E-7 3.61271977222799E-7 1.5387030083413578E-5

GOTERM_MF_DIRECT GO:0004252~serine-type endopeptidase activity 3 0.10695187165775401 0.020965802900630586 C4A, KLKB1, F9 16 255 16881 12.4125 0.7073988828204352 0.4590738338926792 19.007510882150967

GOTERM_BP_DIRECT GO:0006508~proteolysis 3 0.10695187165775401 0.08065829881722537 C4A, KLKB1, F9 17 500 16792 5.926588235294118 0.9999999963425552 0.8845017752795505 66.00343320073763

GOTERM_CC_DIRECT GO:0005886~plasma membrane 5 0.17825311942959002 0.5577325197977883 C4A, KLKB1, F9, HRG, B2M 18 4121 18224 1.2283965596268436 1.0 0.9977989112580559 99.97186965236665

Annotation Cluster 6 Enrichment Score: 2.4192080131395843

Category Term Count % PValue Genes List Total Pop Hits Pop Total Fold Enrichment Bonferroni Benjamini FDR

GOTERM_BP_DIRECT GO:0030449~regulation of complement activation 3 0.10695187165775401 3.645542798934609E-4 CFP, C8B, C4A 17 30 16792 98.7764705882353 0.08077779173927335 0.013939826863130889 0.46669039681971336

GOTERM_BP_DIRECT GO:0006956~complement activation 3 0.10695187165775401 0.003037495016610305 CFP, C8B, C4A 17 87 16792 34.06085192697769 0.5047681083451294 0.09551529097214417 3.8276441472065548

UP_KEYWORDS Immunity 4 0.14260249554367202 0.008863209541506902 CFP, C8B, C4A, B2M 19 500 20581 8.665684210526315 0.5223733307073185 0.07882281261266844 9.09327703269044

UP_KEYWORDS Innate immunity 3 0.10695187165775401 0.02144367197635731 CFP, C8B, C4A 19 261 20581 12.45069570477919 0.8345663438481173 0.15088638186771475 20.71576499658161
